# The cardinal technique for symmetric reorientation during bicuspid aortic valve-sparing root replacement

**DOI:** 10.1016/j.xjtc.2025.10.011

**Published:** 2025-10-25

**Authors:** Albert J. Pedroza, Alexander K. Reed, Y. Joseph Woo

**Affiliations:** a Department of Cardiothoracic Surgery, Stanford University School of Medicine, Palo Alto, Calif; b Department of Bioengineering, Stanford University, Palo Alto, Calif

**Figure:**
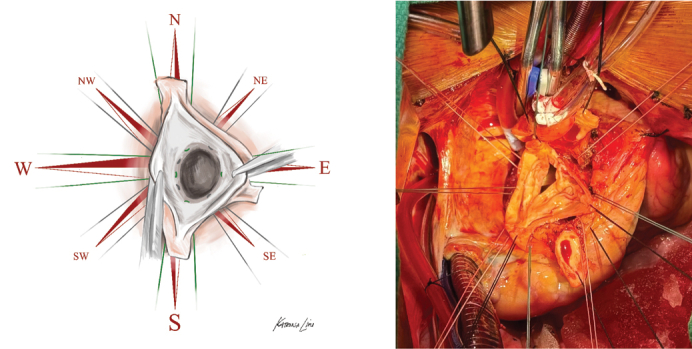
Cardinal subannular suture technique for bicuspid aortic valve-sparing root replacement.

**Central Message**

Standardized subannular suture placement technique enables symmetric orientation of bicuspid aortic valves during reimplantation valve-sparing root replacement.

Valve-sparing aortic root replacement (VSARR) with reimplantation is an adaptable technique for repair of aortic root aneurysms and regurgitant aortic valves. Reimplantation of trileaflet aortic valves is predicated on maintenance of inherent symmetric sinus orientation (120°/120°/120°) to optimize leaflet coaptation.^1^ With respect to subannular sutures, varying technical reports describe symmetric, triangulated configurations, including beneath each leaflet nadir only (3 sutures),^2^ beneath both commissures and nadirs (6 sutures),^3^ and beneath commissures plus 3 evenly spaced sutures in each sinus (12 sutures).^4^ In contrast, inherently asymmetric commissures in patients with bicuspid aortic valve (BAV) have led to multiple solutions, including reorientation into symmetric 180°/180° configurations^5^ and commitment to slightly asymmetric^E1^ 170°/190° and very asymmetric^E2^ 150°/210° configurations. These approaches have been described with asymmetric and/or tailored, bespoke subannular suture distributions corresponding to the relative geometry of each cusp, limiting broad applicability along the continuous spectrum of BAV.

Here, we describe a simplified, reproducible approach to BAV VSARR in which symmetric (180°/180°) orientation is achieved via circumferential subannular sutures corresponding to the 2 true commissures, 2 leaflet midpoints (nadir and/or raphe), and intervening positions (between commissures and midpoints). Once placed through a straight graft, this technique results in 8 subannular sutures ultimately oriented radially 45° apart, resembling the cardinal (north, south, east, and west) and intercardinal (northeast, northwest, southeast, and southwest) directions of an 8-wind compass rose (Figure 1).

**Figure 1:**
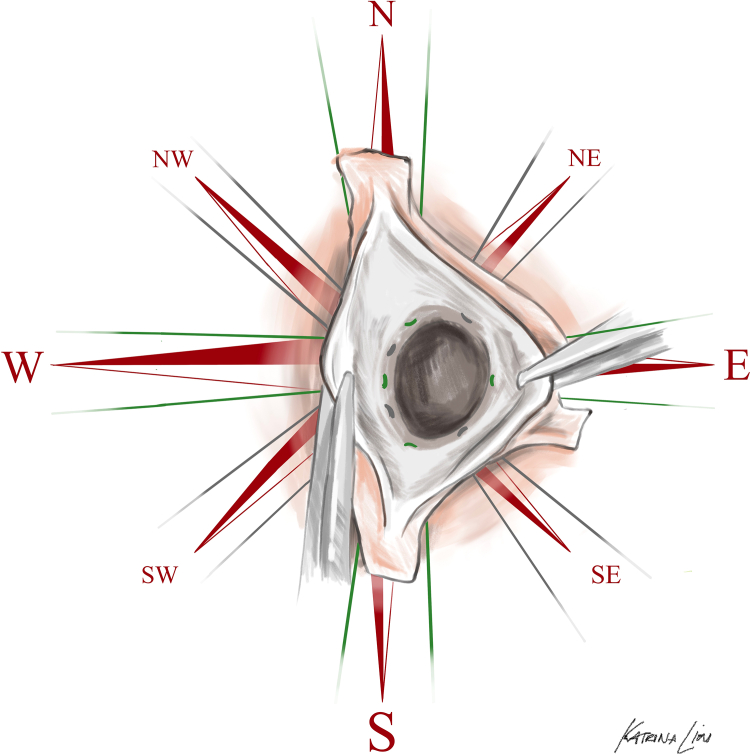
Operative illustration of the cardinal technique with cardinal (*green*) and intercardinal (*grey*) suture placement depicted in a left-right fused bicuspid aortic valve.

This cardinal technique—named in recognition of these navigational points and the red color that serves as both uniform color and team name at Stanford University—enables reproducible, teachable conduct of VSARR for BAVs geared toward optimizing symmetric commissural resuspension and simplifying leaflet repair.

## Technique

**Figure 2:**
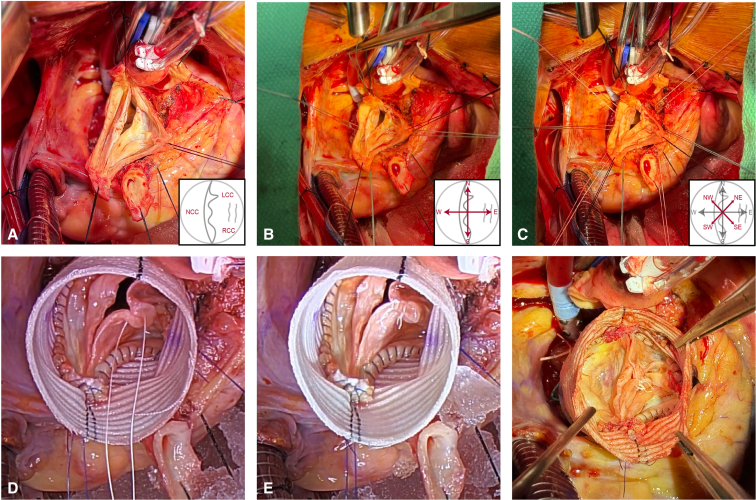
A, Completed aortic root deconstruction in a patient with left-right fused asymmetric bicuspid aortic valve, viewed with patient cranial direction oriented at the top. Inset diagram illustrates leaflet anatomy. B, Placement of 4 (*green*) cardinal sutures. C, Placement of 4 (*white*) intercardinal sutures. D, Completed internal running suture line and free-margin shortening of fused left-right cusps; the righthand photo depicts completed shortening. E, Completed aortic valve reimplantation and repair with symmetric 180°/180° reorientation.

An illustrative case of a 45-year-old asymmetric BAV with left-right leaflet fusion and severe aortic regurgitation is presented to illustrate operative conduct (Video 1). Following cannulation, initiation of cardiopulmonary bypass, and aortic crossclamping, cardioplegia is administered. The aorta is transected and resected to leave a minimal cuff of sinus tissue for reimplantation. Coronary buttons are created and mobilized, followed by deep circumferential root dissection (Figure 2, *A*).

The aortic annulus is approached as a compass face, with the coaptation line oriented in the north-south axis. Subannular sutures of a single color are placed in each of the 4 cardinal directions, beginning in the north, beneath the left noncommissure, and progressing circumferentially with the west beneath the nadir of the noncoronary cusp, south beneath the right noncommissure, and east beneath the left-right cusp raphe (Figure 2, *B*). An additional set of 4 intercardinal (northwest, northeast, southwest, and southeast) sutures of a secondary color are placed at the physical midpoint between each cardinal suture (Figure 2, *C*). The left ventricular outflow tract is sized and a straight Hemashield Platinum polyethylene terephthalate graft (Getinge) 3 mm larger than this diameter is selected. The graft is divided into quadrants by marking midpoints between each black line. Sutures are passed through the graft to recapitulate annular coronet structure, with commissural (north/south) sutures placed 5 rings (approximately 10 mm) from the base, intercardinal sutures placed 3 to 4 rings (6-8 mm) from the base, and the west suture placed 2 rings (4 mm) from the base. The east suture is placed 3 rings (6 mm) from the base to respect the intermediate height of the raphe without elevating the fused leaflet excessively. The graft is seated and sutures tied at approximating tension.

Next, commissures are resuspended high at each black line on the polyethylene terephthalate graft to complete the 180°/180° reorientation. The noncoronary cusp, which will serve as the reference leaflet for subsequent leaflet repair, is assessed to ensure adequate height. The fused left/right sinus, which will necessarily be redundant from reduction to 180° orientation, is reimplanted into the graft with running 4-0 polypropylene suture, taking care to distribute excess sinus tissue length appropriately to avoid distorting the conjoined leaflet. The noncoronary sinus is then reimplanted into the graft. Aortic valve leaflet repair is then undertaken via free margin shortening of the fused leaflet with plication sutures of CV-6 Gore-Tex (W.L. Gore & Associates) (Figure 2, *D*). Raphe incision may be necessary to allow adequate leaflet motion but was not required in this case. Following leaflet repair optimization, the left coronary button is reimplanted in standard fashion with 5-0 polypropylene sutures. The aortic valve repair is then pressure tested with cold blood perfusion into the root graft with right angle clamps placed on the distal end and left ventricular vent disconnected. Right coronary button and graft-to-aorta anastomosis are performed to complete the repair (Figure 2, *E*). Following reperfusion and weaning from bypass, aortic valve leaflet geometry and competence are assessed with transesophageal echocardiography. In the described case, echocardiography demonstrated 10 mm of coaptation height, no residual aortic regurgitation, and peak and mean gradient of 14 and 7 mm Hg, respectively. The patient was extubated on the day of surgery, transitioned to floor status on postoperative day 2, and discharged home on postoperative day 5 with no immediate complications.

## Discussion

Multiple effective technical strategies have been reported for reimplantation of asymmetric BAV during VSARR operations. Compared with other 180°/180° reorientation techniques, the cardinal technique simplifies subannular suture placement by prescribing 8 sutures placed at defined locations relative to the true commissural and anticommissural (eg, nadir and raphe) positions. This removes the inherent variability of the number of sutures required when determined by the relative circumference of each leaflet. By setting these cardinal points, suture placement through the VSARR graft is further simplified by dividing the circumference into quadrants and spacing the 8 sutures evenly around the ring. It is critical to post the commissures high and centered on the prefabricated markings to achieve full symmetric reorientation. In regurgitant valve cases, this approach simplifies leaflet repair by setting the height of the reference (nonfused) cusp to which the prolapsing leaflet must reach. One consequence of this technique relative to the 180°/180° technique described by Jahanyar and colleagues,^5^ or asymmetric 170°/190°/150°/210° techniques^E1^^,^^E2^ is an overall reduced number of subannular stitches, which might reduce stabilization effect of very dilated annuli. Furthermore, commitment to 180°/180° orientation (rather than asymmetric) produces greater redundancy of the cuff aortic tissue on the fused leaflet that must be managed with proper spacing of the running hemostatic suture line.

With respect to graft sizing, we generally use a straight Hemashield Platinum graft sized 3 mm larger than the native left ventricular outflow tract, generally a 28- to 32-mm graft. In patients with aneurysm, this allows for improved leaflet coaptation without crowding following reimplantation while providing some annular stabilization by tying subannular sutures at approximating tension. In cases of very dilated annuli, this may be adjusted to true size the graft to the native left ventricular outflow tract, whereas a left ventricular outflow tract +5 mm choice may be advisable when VSARR is performed primarily as a valve repair strategy in a small annulus, and annuloplasty sutures may be tied over a cone sizer to prevent excessive annuloplasty effect.

Although presented in a left-right fusion case, we have found that this technique is easily applied to a Sievers type 0 case (no raphe) or adapted to less common fusion morphologies with varying degrees of asymmetry. This technique may not be practical in severely asymmetric cases, in which the nonfused leaflet may be of insufficient size and height to accommodate 180° orientation. Thus, for BAV with commissural angle <140°, we advocate for consideration of an asymmetric reimplantation strategy such as a 170°/190° or even 150°/210° reorientation, depending on the relative size of the nonfused leaflet and intraoperative assessment of coaptation.

## Conflict of Interest Statement

The authors reported no conflicts of interest.

The *Journal* policy requires editors and reviewers to disclose conflicts of interest and to decline handling or reviewing manuscripts for which they may have a conflict of interest. The editors and reviewers of this article have no conflicts of interest.

## Supplementary Data

Video 1 Case presentation and operative video. Video available at: https://www.jtcvs.org/article/S2666-2507(25)00470-5/fulltext.
