# Supplementary figures and images for: The cardinal technique for symmetric reorientation during bicuspid aortic valve-sparing root replacement
Source: JTCVS Tech. 2025 Oct 25;35:102135. doi: 10.1016/j.xjtc.2025.10.011 (PMC12881770; doi:10.1016/j.xjtc.2025.10.011)

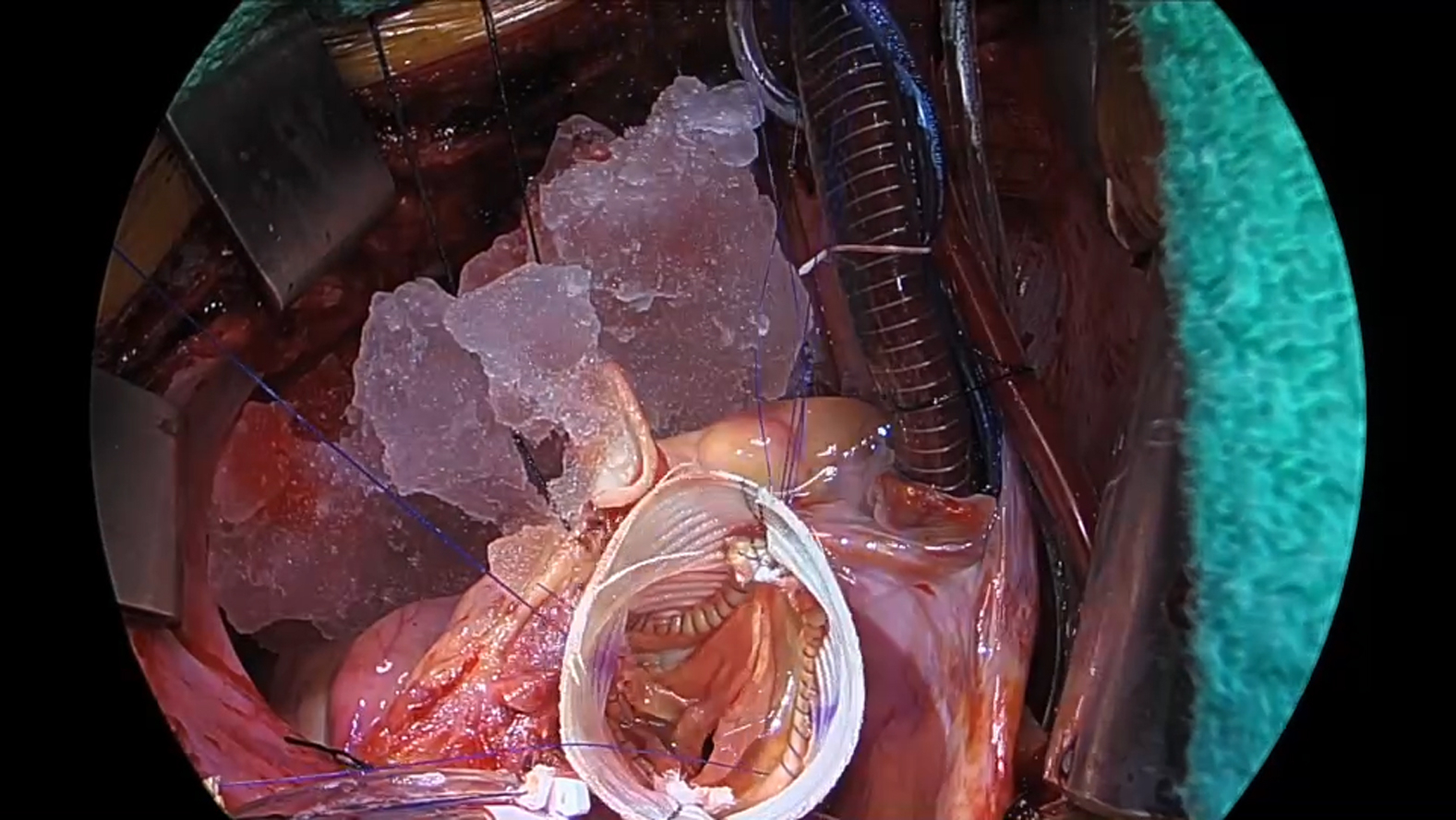

Supplement: Video 1 — Case presentation and operative video. Video available at: https://www.jtcvs.org/article/S2666-2507(25)00470-5/fulltext. [file fx2.jpg]
